# Clinical prediction models for post-stroke depression: a systematic review and meta-analysis

**DOI:** 10.3389/fpsyt.2025.1629023

**Published:** 2025-12-17

**Authors:** Honggang Wu, Yating Xiao, Xin Hu, Chao You, Niandong Zheng, Lu Ma

**Affiliations:** 1 Department of Neurosurgery, West China Hospital, Sichuan University, Chengdu, China; 2 Department of Neurosurgery, West China Hospital, Sichuan University/West China School of Nursing, Sichuan University, Chengdu, China; 3 Department of Cerebrovascular Disease, The People’s Hospital of Leshan, Leshan, Sichuan, China

**Keywords:** post-stroke depression, machine learning models, PROBAST+AI, artificial intelligence, depression prediction, meta-analysis, systematic review

## Abstract

**Background:**

Post-stroke depression (PSD) is a prevalent neuropsychological consequence of stroke, associated with cognitive decline, disability, and increased mortality. Early prediction of PSD is critical for timely interventions and better outcomes. This study evaluates the effectiveness of various clinical prediction models, particularly machine learning methods, in forecasting PSD.

**Methods:**

A systematic review and meta-analysis were conducted to evaluate predictive models for post-stroke depression (PSD) using data from 16 studies. The databases searched included PubMed, Embase, Cochrane Library, and Web of Science, covering publications from the year 2000 to the present. The risk of bias in the predictive models was assessed using the Prediction model Risk of Bias Assessment Tool+AI (PROBAST+AI). The review encompassed both traditional statistical methods and machine learning algorithms. Predictive accuracy was analyzed through the area under the curve (AUC) values of these models, considering various data sources, such as clinical, cognitive, and biomarker data. R packages, including ‘metafor,’ ‘meta,’ and ‘forestplot,’ were used for the analysis.

**Results:**

16 studies were included. Neural Network models yielded the highest pooled AUC (0.88, 95% CI: 0.45–0.98), although this estimate was based on only two studies and exhibited wide confidence intervals. Logistic Regression, Decision Tree, and K-Nearest Neighbor models showed comparable predictive ability, with pooled AUC values ranging from 0.77 to 0.83. Support Vector Machine models demonstrated the lowest predictive performance (AUC = 0.68) but exhibited the lowest heterogeneity. Among different data sources, functional, physical, and cognitive assessments yielded the highest predictive accuracy (AUC = 0.86, 95% CI: [0.81, 0.90]), followed by biomarker-based models (AUC = 0.80, 95% CI: [0.71, 0.86]). Within retrospective studies, biomarker-based data sources demonstrated significantly superior predictive performance (AUC = 0.94, 95% CI: 0.92–0.96).

**Conclusions:**

Machine learning models, particularly Neural Networks, show potential for predicting post-stroke depression, although current evidence is limited by small sample sizes and high heterogeneity. Traditional approaches such as Logistic Regression and Decision Tree models also demonstrate stable and competitive performance. Among data sources, functional, physical, and cognitive assessments provide the strongest predictive value, while biomarker-based models appear particularly effective in retrospective analyses. Despite these findings, the limited number of high-quality studies and methodological inconsistencies highlight the need for rigorous, prospective, and multicenter validation to establish reliable and generalizable predictive models for post-stroke depression.

**Systematic Review Registration:**

https://www.crd.york.ac.uk/prospero/, identifier CRD42025635227.

## Introduction

Depression is a frequent and severe neuropsychological consequence of stroke, strongly linked to cognitive decline, higher levels of disability, and increased risk of death (1–3). Post-stroke depression (PSD) may arise weeks, months, or even years after the onset of a stroke. Its progression is dynamic, with higher rates observed in the acute phase and a gradual decline over time. The incidence of early-onset PSD (occurring within two weeks of stroke) varies between 17.6% and 59.2%, likely due to differences in study populations, PSD definitions, and evaluation methods (4–7). One meta-analysis showed that a hazard ration for PSD and all-cause mortality was 1.59 (3). Hence, the early prediction of the risk of developing PSD holds crucial clinical significance, as it can assist healthcare professionals in assessing a patient’s condition and planning timely interventions. However, it remains unclear which clinical prediction model and data source offer the best performance for this purpose.

In recent years, the advancement of clinical prediction models, including machine learning, has been extensively applied in personalized medicine and decision support systems (8). For example, a recent study showed machine learning analysis successfully demonstrated the ability to predict clinical outcomes after first-ever ischemic stroke and identified the leading prognostic factors that contribute to this prediction (9).

Numerous studies have successfully demonstrated the ability of these models, particularly those incorporating machine learning, to accurately predict specific clinical outcomes following a stroke. Research on clinical prediction models for Post-Stroke Depression (PSD) has explored various model types, including Decision Trees, XGBoost, and Random Forest, as well as data sources such as sociological data and liver function test (7, 9–12). the predictive performance among these different models has not been fully compared. Therefore, we carried out this systematic review and meta-analysis to estimate the clinical prediction models for PSD.

## Methods

This systematic review was conducted in accordance with the guidelines outlined in the Preferred Reporting Items for Systematic Reviews and Meta-Analyses (PRISMA 2020) statement (13). A protocol was not prepared.

### Information sources and search strategy

For this systematic review and meta-analysis, we conducted a comprehensive and systematic search across four major databases: PubMed, Embase, Cochrane Library, and Web of Science (Publication year: 2000-present). The final search was conducted on January 5, 2025. Our search strategy combined subject headings and free-text keywords, ensuring no restrictions on region or language. The search terms were specifically designed to include keywords and medical subject headings (MeSH) related to post-stroke depression, clinical prediction models, and meta-analysis. The search results were refined and combined using the “AND” logical operator to create the final retrieval dataset. Detailed information on the search strategy of Pubmed was shown as example, and was provided in **Supplementary Table S1**.

We used the PICOTS system for the systematic review, which allows framing of the review’s aim, search strategy, and study inclusion and exclusion criteria, as described below.

P (Population): Patients with PSD.

I (Index models): Clinical prediction models including machine learning models and Logistic Regression.

C (Comparator): Other Clinical prediction models.

O (Outcome): Prediction accuracy for PSD.

T (Timing): The outcome was predicted after evaluating basic information upon admission, clinical scoring scale results, and laboratory indicators.

S (Setting): The intended use of the risk prediction models was to individualize the prediction of PSD.

### Inclusion and exclusion criteria

#### Inclusion criteria

Studies were included if they focused on patients with stroke, including ischemic stroke, hemorrhagic stroke, or mixed stroke types, and utilized prediction models (traditional statistical methods or machine learning) to predict the occurrence of PSD. Eligible studies included cohort studies, case-control studies, case-cohort studies, and nested case-control studies. Models could focus on predicting PSD at any time point (acute, subacute, or chronic phases), and studies without independent validation datasets were also included. Additionally, studies utilizing machine learning approaches published on the same dataset were eligible, provided they reported at least one model performance metric, such as ROC, AUC, C-index, sensitivity, specificity, accuracy, calibration curves, or F1 score. Only original studies published in English were included, regardless of whether the exact timing or classification of PSD onset was specified.

#### Exclusion criteria

Studies were excluded if they were meta-analyses, reviews, guidelines, editorials, or expert opinions, or if they solely analyzed risk factors without developing or validating prediction models. Research lacking essential predictive accuracy indicators, such as ROC, C-index, sensitivity, specificity, or calibration curves, was excluded, as were studies with small sample sizes (<50 cases) or insufficient effective events (EPV < 10). Studies focusing primarily on the validation of assessment scales, the prediction of single factors, case series, case reports, randomized controlled trials, descriptive studies, or research related to pediatric populations (patients under 18 years of age) were also excluded.

### Study selection and data extraction

To ensure the integrity of the data, H.W. and Y.X. independently conducted the data extraction process. Also, PROBAST+AI tool was applied independently by the two authors. Discrepancies were resolved through discussion or by consulting L.M. In detail, The retrieved articles were imported into EndNote X9, and duplicate records were removed. Titles and abstracts were reviewed to exclude studies that did not meet the eligibility criteria. Full-text articles were then downloaded and assessed to identify studies eligible for inclusion in the systematic review. If studies reported missing summary statistics (such as missing values for AUC, sensitivity, or specificity), the reviewers attempted to contact the authors to request the missing data. If contacting authors was not feasible, studies with missing essential information were excluded from the analysis. The key characteristics of the included studies are summarized in **Supplementary Table S2**.

### Quality assessment and publication bias

We assessed the risk of bias in predictive models using the Prediction model Risk of Bias Assessment Tool +AI (PROBAST+AI) (14). PROBAST+AI is an updated tool for evaluating the quality, risk of bias, and applicability of prediction models, including AI/machine learning models. It follows four steps: defining the model’s purpose using PICOTS criteria, classifying the study type (model development, evaluation, or both), assessing quality or risk of bias for each domain (e.g., study design, predictors, outcomes), and making an overall judgment on the model’s quality, risk of bias, and applicability. This tool improves upon PROBAST-2019 and can be applied to both traditional and AI-based models, helping in the critical appraisal of predictive modeling studies. The publication bias were evaluated by Begg test.

### Data synthesis and statistical analyses

We conducted a meta-analysis to evaluate the AUC indicators of predictive models, including SVM, Decision Tree, Logistic Regression, Neural Network, K-nearest Neighbor. We also evaluate the AUC indictors of different data source including sociological data and clinical data, liver function test, Functional, physical and cognitive tests. A random-effects model was prioritized for the meta-analysis of AUC. The analysis was performed using R version 4.2.0 (R Development Core Team, Vienna, http://www.R-project.org). The R packages employed for this process included ‘metafor,’ ‘meta,’ and ‘forestplot.’ The possible causes of heterogeneity among study results will be explored by subgroup analysis. A sensitivity analysis was performed using the leave-one-out method.

## Results

### Study characteristics

**Figure 1:**
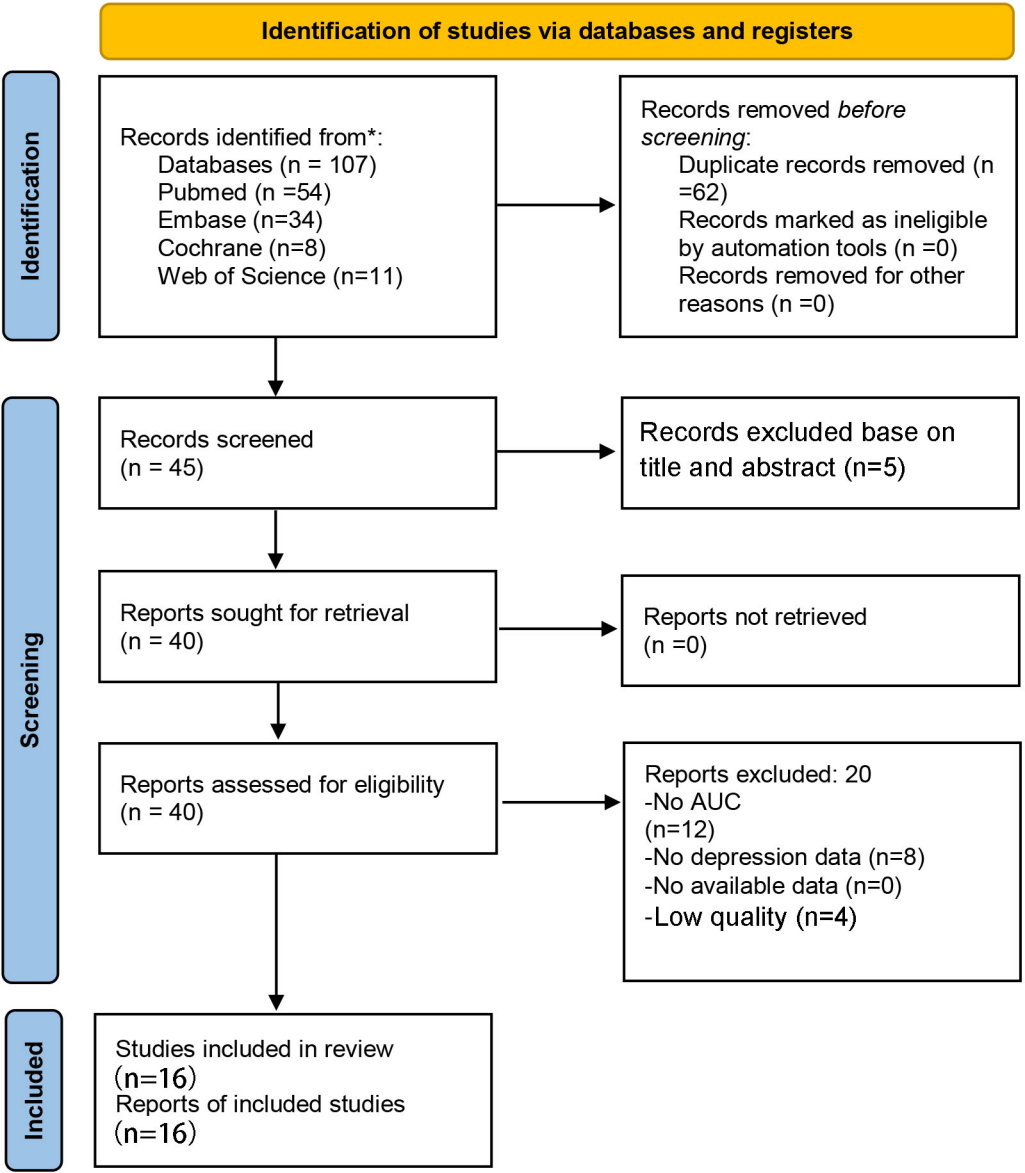
PRISMA flow diagram depicting the study selection process. This diagram illustrates the identification, screening, eligibility, and inclusion of studies in the systematic review and meta-analysis.

Major data sources were PubMed, Embase, Cochrane, and Web of Science databases. The literature screening procedure is shown in **Figure 1**. There were 20 studies included in this meta-analysis, which incorporated both machine learning models and the Logistic Regression Model. Specifically, the machine learning models included Support Vector Machine (SVM) (9, 15, 16); Gradient Boosting (9), Light Gradient Boosting (LightGBM) (17, 18), Extreme Gradient Boosting (XGBoost) (15, 17–19), CatBoost (15, 17, 18), Gradient Boosting Decision Tree (GBDT) (15, 17), Random Forest (15–17, 20), AdaBoost (17), decision tree (17), Artificial Neural Network (ANN) (21), Log-Linearized Gaussian Mixture Network (22) and K-nearest neighbor (16); and linear regression methods and extensions such as Stepwise Multiple Linear Regression (22)and Partial Least Squares (PLS) regression (22). Logistic regression model included logistic regression analysis (12, 17, 18, 22–30), binary logistic regression analysis (31) and multivariate logistic regression model (32).

In these included studies, multiple data sources were utilized to predict the occurrence of PSD. According to the data sources, we categorized the articles into five groups: sociological and clinical data (3, 9, 12, 17, 19, 23, 25–27, 29, 32, 33), liver function tests (15), functional, physical, and cognitive assessments (16, 18, 21, 22), and biomarkers (17, 20, 24, 25, 28, 30, 31, 33). The detail of included studies were listed in **Supplementary Table S2**.

### Risk of bias in included studies

The risk of bias in prediction models was assessed using the PROBAST tool (34), with a focus on four key domains: predictors, participants, outcomes, and analysis. The detailed findings from the assessment are presented in **Supplementary Table S3**. Five studies (highlighted in red) were excluded due to unclear risk of bias and applicability concerns. The result of the Begg test (z = 0.87, p-value = 0.3847; Bias estimate = 77.0000, SE = 88.5818) indicates that there is no statistically significant evidence of publication bias.

### Meta-analysis

**Figure 2:**
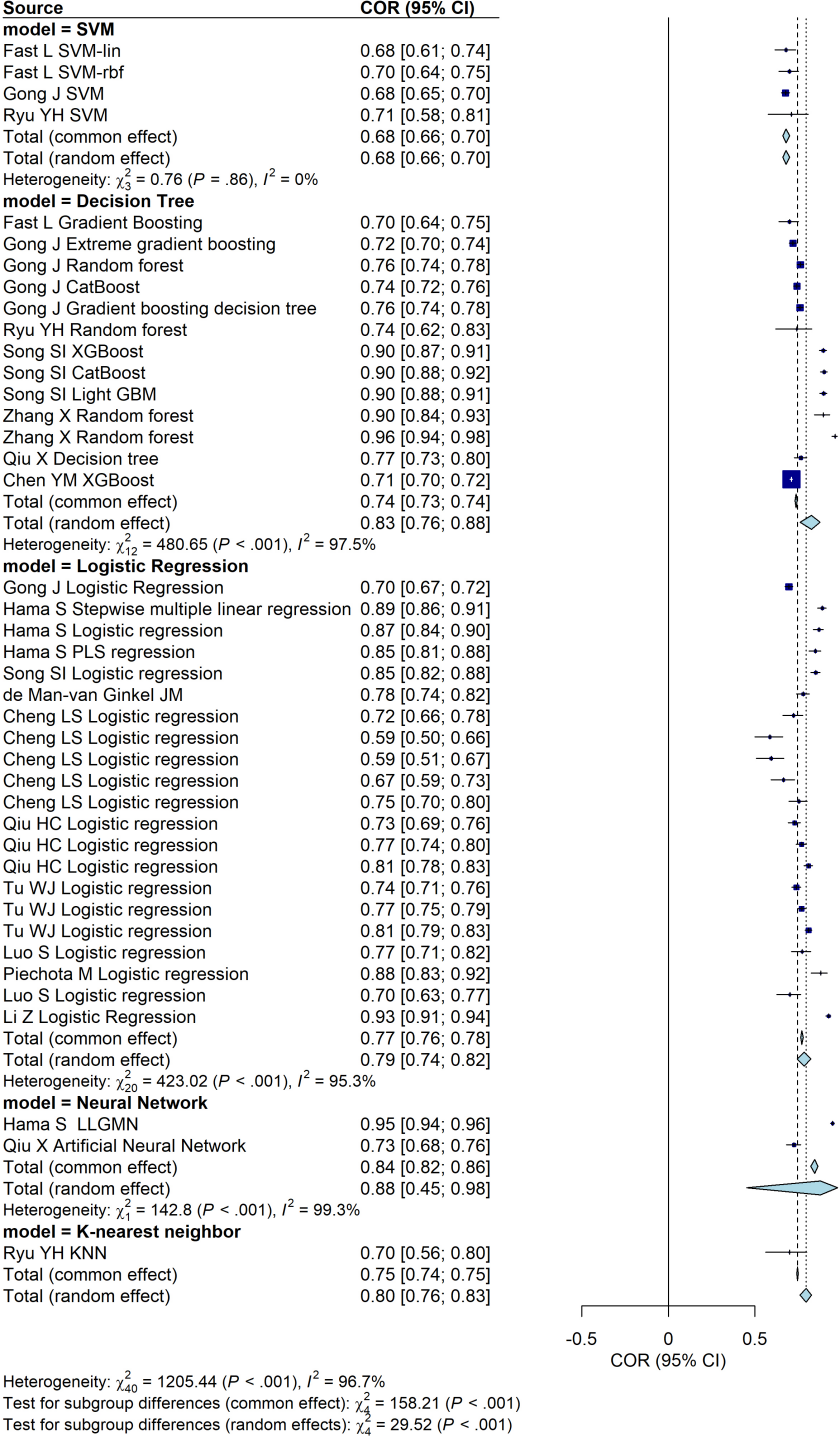
Forest plot of the predictive performance of different models for PSD. The plot shows area under the curve (AUC) values with their 95% confidence intervals for each included study.

We first compared the predictive performance of different models using a random-effects model. The analysis revealed the following AUC values with corresponding 95% confidence intervals (CI): the Support Vector Machine (SVM) group achieved an AUC of 0.68 (95% CI: [0.66, 0.70]), the Decision Tree group had an AUC of 0.83 (95% CI: [0.76, 0.88]), the Logistic Regression group showed an AUC of 0.79 (95% CI: [0.74, 0.82]), the Neural Network group achieved the highest AUC of 0.88 (95% CI: [0.45, 0.98]), and the K-Nearest Neighbor group demonstrated an AUC of 0.80 (95% CI: [0.76, 0.83]). Among these models, the Neural Network group appeared to demonstrate the highest predictive performance based on the AUC, although this finding was derived from only two studies and was accompanied by wide confidence intervals. Logistic Regression, Decision Tree, and K-Nearest Neighbor models demonstrated comparable performance, with AUC values ranging from 0.77 to 0.78. In contrast, the SVM group showed the lowest predictive performance with an AUC of 0.68. However, the SVM group have the lowest I^2^. These findings suggest that Neural Network models may provide superior predictive accuracy, while traditional models such as Logistic Regression and Decision Trees also perform well in this context (**Figure 2**). The stability and generalizability of this result still need further verification due to the limited number of studies included and the significant heterogeneity observed.

**Figure 3:**
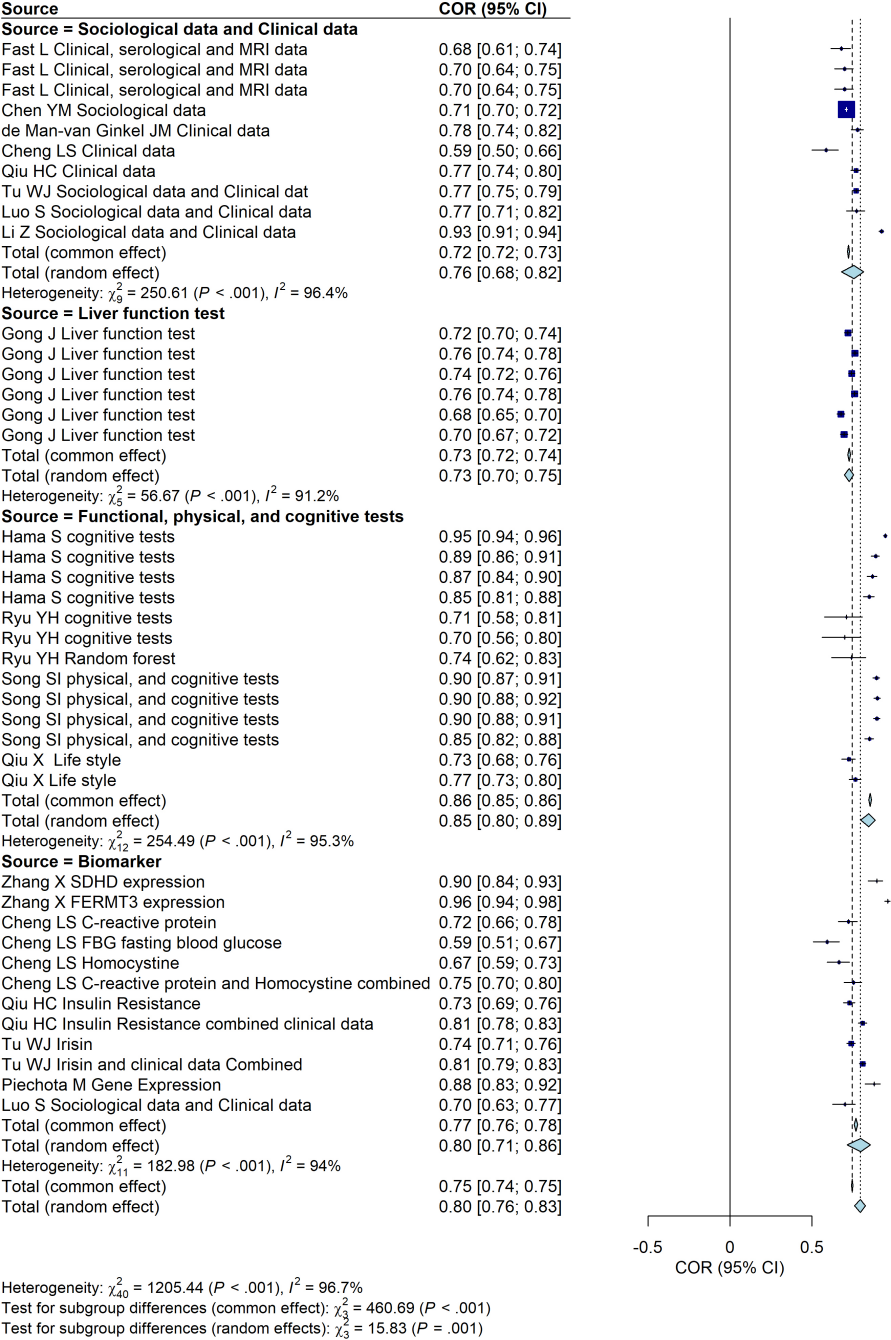
Forest plot of the predictive performance of different data sources for PSD. The plot shows area under the curve (AUC) values with their 95% confidence intervals for each included study.

We then a comprehensive analysis of the predictive performance of models for PSD based on different data sources, including sociological and clinical data, liver function tests, functional, physical, and cognitive assessments, and biomarkers. The predictive performance of each data source group is expressed through AUC values and their corresponding 95% confidence intervals (CI), with heterogeneity analysis conducted. A random-effects model is preferred for the subgroup analysis. The results indicate the following predictive performances: the sociological and clinical data group (AUC = 0.76, 95% CI: [0.68, 0.82]), the liver function test group (AUC = 0.73, 95% CI: [0.70, 0.75]), the functional, physical, and cognitive assessment group (AUC = 0.86, 95% CI: [0.85, 0.86]), and the biomarker group (AUC = 0.80, 95% CI: [0.71, 0.83]). Moreover, these findings highlight the variations in predictive performance across different data sources, with the functional, physical, and cognitive assessment group showing the highest AUC, indicating superior predictive ability (**Figure 3**).

#### Stratified meta-analysis

**Figure 4:**
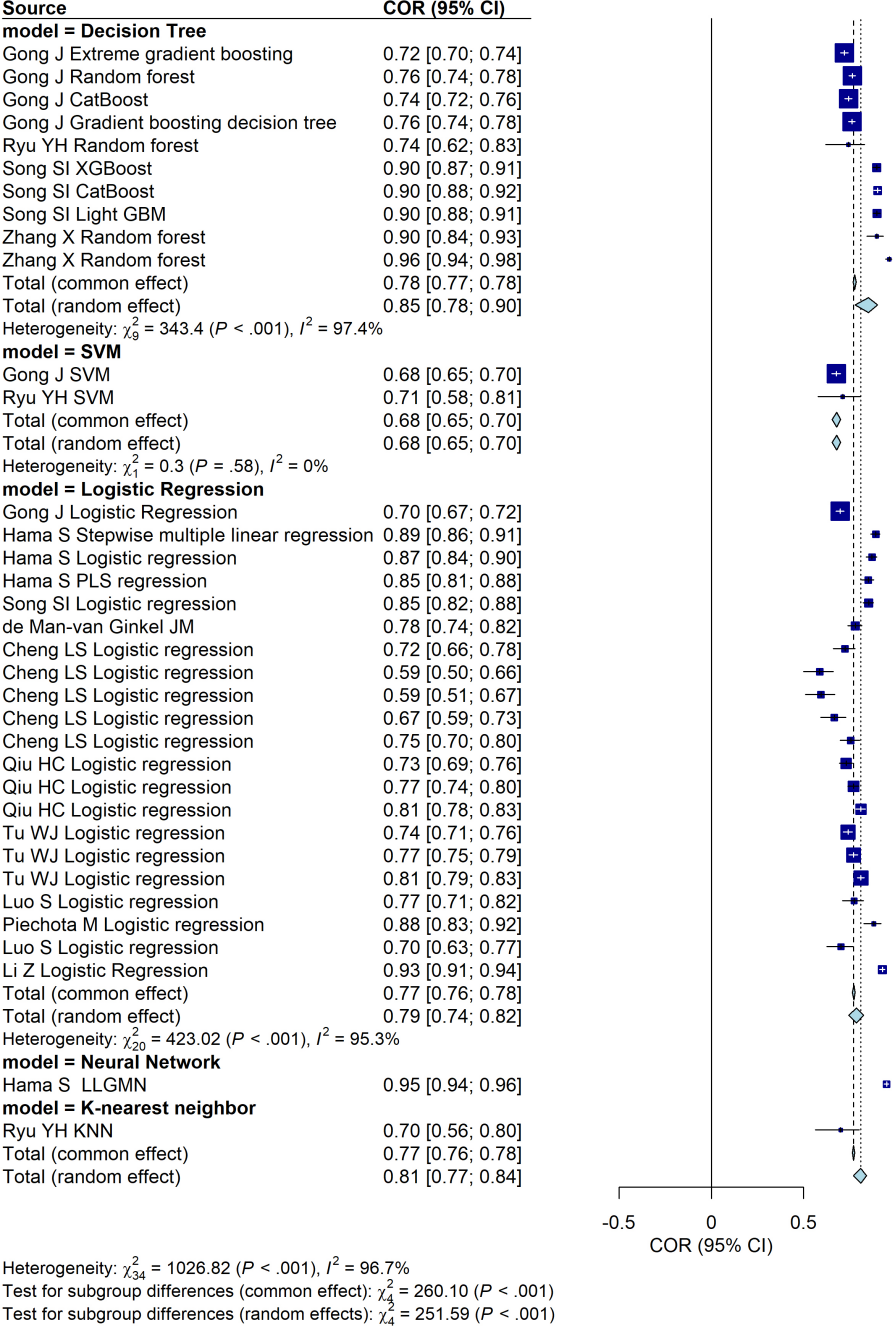
Forest plot illustrating the predictive performance of different models for PSD (excluding studies involving new-onset stroke). The plot presents the area under the curve (AUC) values along with their 95% confidence intervals for each included study.

**Figure 5:**
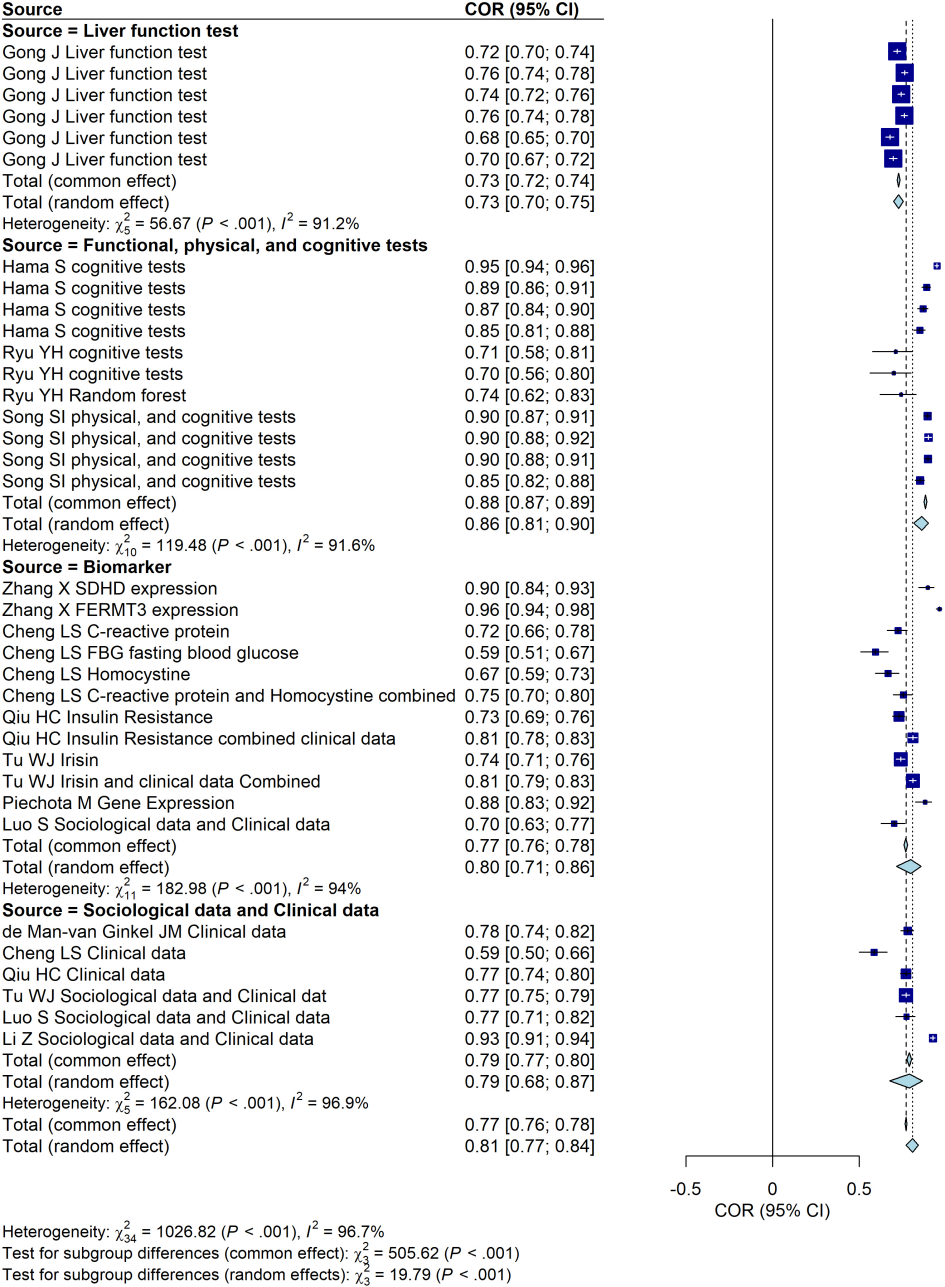
Forest plot illustrating the predictive performance of different data sources for PSD (excluding studies involving new-onset stroke). The plot presents the area under the curve (AUC) values along with their 95% confidence intervals for each included study.

In the included studies, there were three studies (9, 19, 21), involved patients with new-onset stroke. These studies were excluded from the subsequent analyses. Among the remaining models, the Neural Network group demonstrated the highest predictive performance based on AUC, although this result was derived from a single study. The model with the second-highest predictive performance was the Decision Tree (**Figure 4**). Similarly, after excluding the three studies mentioned above, we evaluated the predictive performance of models for PSD across different data sources and found no statistically significant differences among the groups (**Figure 5**).

**Figure 6:**
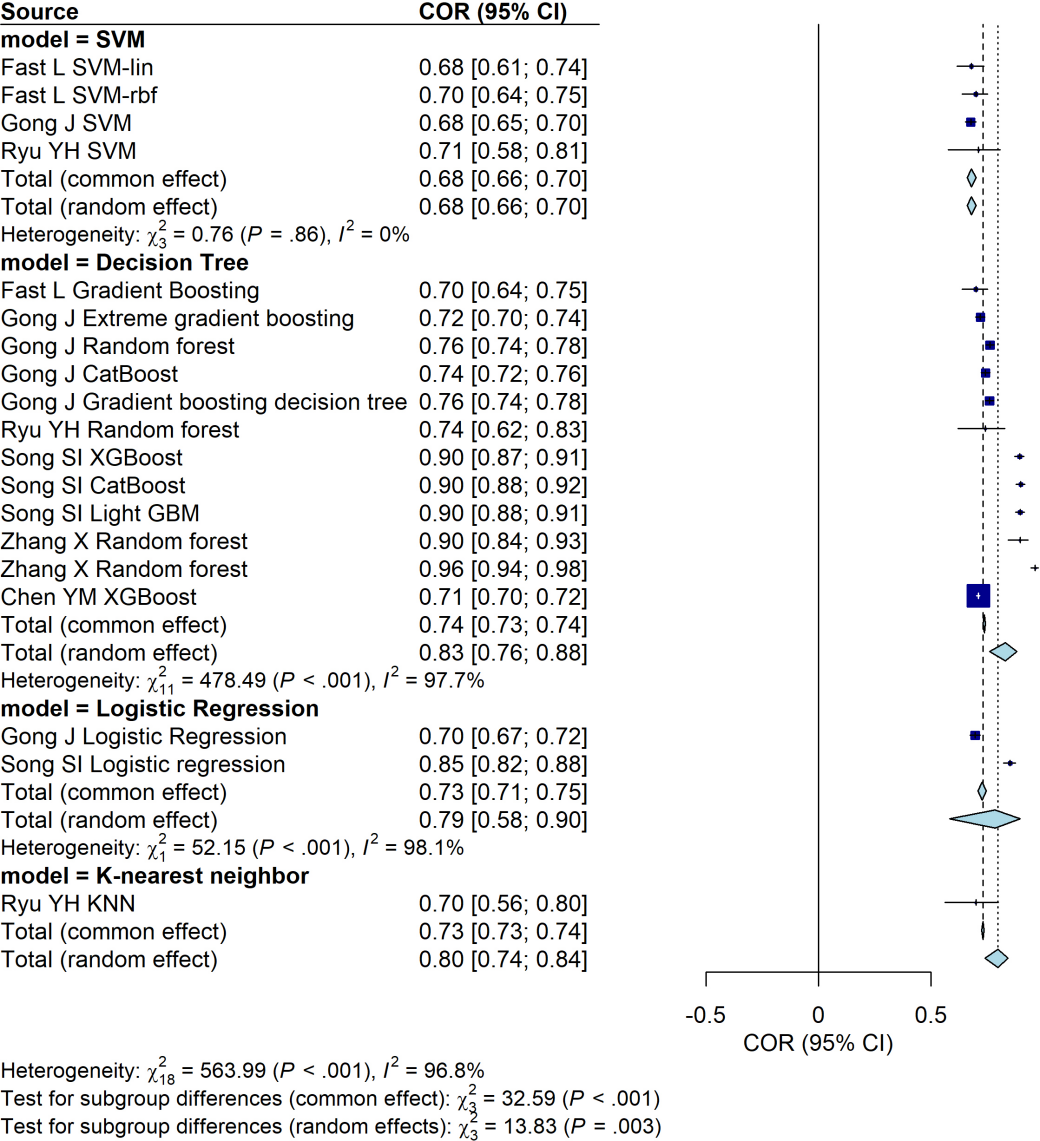
Predictive performance of different model types in retrospective studies. The plot shows area under the curve (AUC) values with their 95% confidence intervals for each included study.

**Figure 7:**
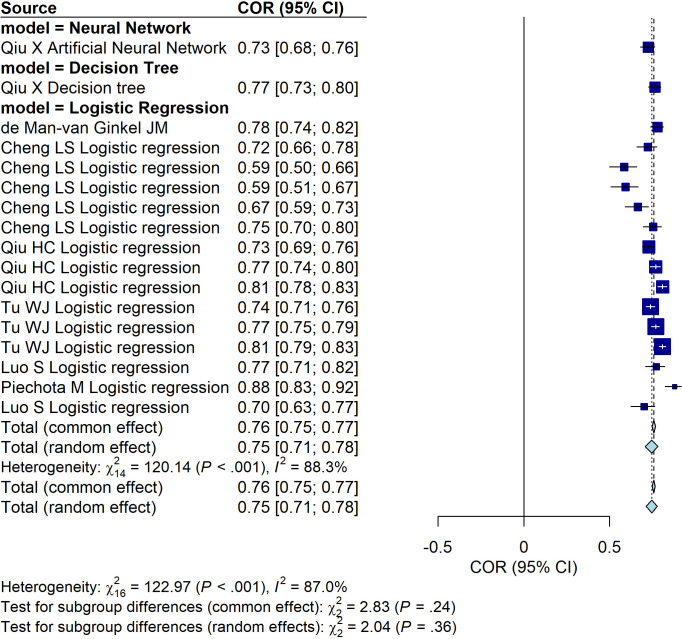
Predictive performance of different model types in prospective studies. The plot shows area under the curve (AUC) values with their 95% confidence intervals for each included study.

**Figure 8:**
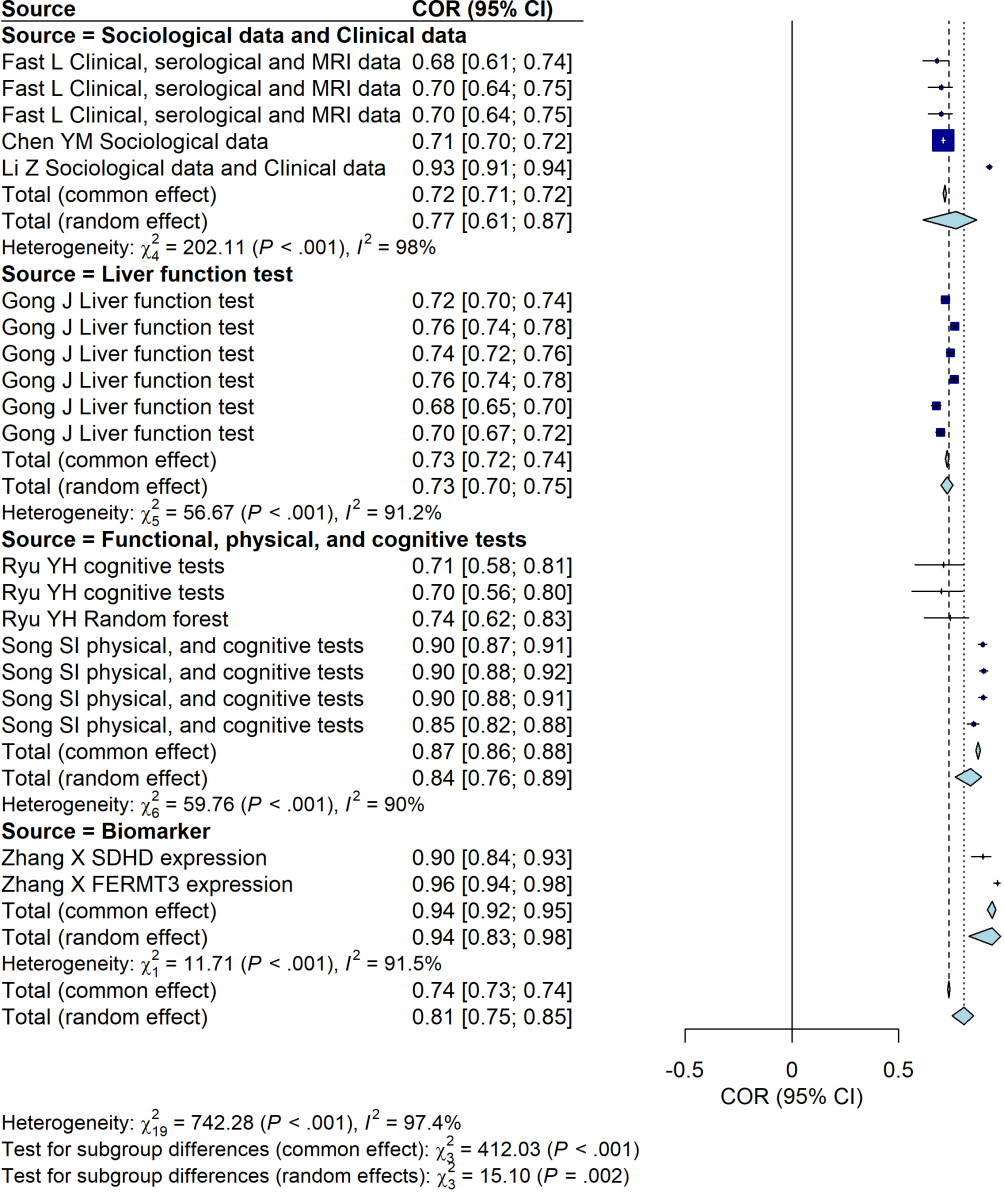
Predictive performance of different data source groups in retrospective studies. The plot shows area under the curve (AUC) values with their 95% confidence intervals for each included study.

**Figure 9:**
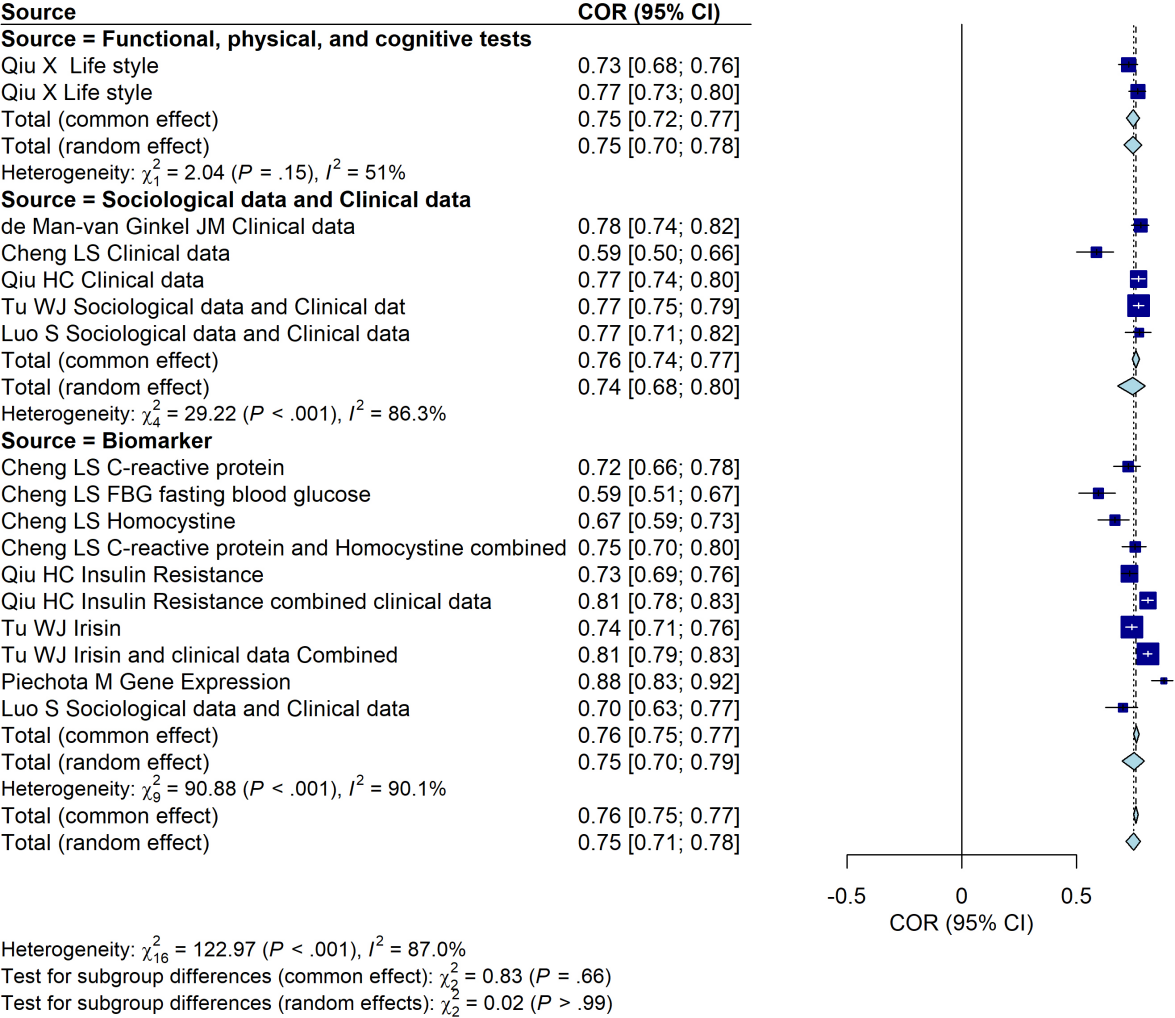
Predictive performance of different data source groups in prospective studies. The plot shows area under the curve (AUC) values with their 95% confidence intervals for each included study.

Next, we categorized all included studies into retrospective and prospective designs and excluded the cross-sectional study (22). We then analyzed the predictive performance of PSD models based on study design (retrospective vs. prospective) and data source (retrospective vs. prospective). In the retrospective studies, no statistically significant differences were observed among the model groups (**Figure 6**). Similarly, in the prospective studies, no statistically significant differences were found among the model groups (**Figure 7**). In the retrospective studies, the biomarker-based data source group demonstrated the best predictive performance, with a common-effect AUC of 0.94 (95% CI: [0.92, 0.96]), showing a slight but notable statistical difference (**Figure 8**). However, in the prospective studies, no statistically significant differences were observed among the data source groups (**Figure 9**).

## Discussion

This meta-analysis compared the predictive performance of various machine learning models and data sources for PSD using a random-effects model. The findings revealed significant variations in predictive accuracy across different models, highlighting both the strengths and limitations of current methodologies. This study may provide evidence for clinicians to choose the proper model or data source to predict PSD. To the best of our knowledge, this is the first meta-analysis combining AI and traditional models in predicting PSD.

Usually, AUCs of 0.5–0.7 were considered with low diagnostic accuracy, 0.7–0.9 were considered with moderate accuracy, and >0.9 indicates high accuracy. The comparison of machine learning models demonstrated that neural networks achieved the highest predictive performance with an AUC of 0.88 (95% CI: [0.94, 0.96]) under the random-effects model, suggesting its potential as a superior predictive tool for PSD. Despite the promising results of neural networks, the limited number of studies and study type (cross-sectional study) underscores the need for further research to enhance the stability and generalizability of these models.

In contrast, the Support Vector Machine (SVM) group exhibited the lowest predictive performance (AUC = 0.68, 95% CI: [0.66, 0.70]), suggesting limitations in its application to PSD prediction. Interestingly, the SVM groups showed a low heterogeneity (I^2^ = 0%), indicating that the studies included in this subgroup produced very similar effect sizes, suggesting consistency in the SVM model’s performance across different studies.

These findings highlight the importance of selecting appropriate machine learning models for PSD prediction, with neural networks demonstrating the most potential but requiring further refinement and validation.

Regarding data sources, the functional, physical, and cognitive assessment group demonstrated the highest predictive performance (AUC = 0.86, 95% CI: [0.85, 0.86]), emphasizing its critical role in predicting PSD. This strong performance may stem from the direct relationship between functional and cognitive impairments and PSD. Biomarker-based studies, while less predictive overall (AUC = 0.77, 95% CI: [0.76, 0.78]), revealed the potential value of inflammatory and metabolism-related indicators as predictors of depression. These findings further underscore the importance of exploring inflammatory and metabolic biomarkers in PSD prediction. Future research should focus on identifying novel biomarkers that can serve as tools for early screening and prediction of PSD, which may improve the efficiency and accuracy of clinical interventions.

In the stratified meta-analysis, the biomarker-based data source group in retrospective studies demonstrated a significantly higher predictive performance, with a common-effect AUC of 0.94 (95% CI: 0.92–0.96), indicating a slight but notable statistical advantage over other data source groups (**Figure 8**). Regarding validation methods, all machine learning studies employed internal cross-validation, whereas none of the regression-based studies used cross-validation. Only one study applied nested cross-validation (9), however, its AUC was relatively low.

### Limitations

This study has several limitations. The number of studies evaluating the use of machine learning for PSD prediction is relatively small, which may limit the generalizability and robustness of the findings. Additionally, differences in participant characteristics, such as age, comorbidities, and stroke severity, may have contributed to the observed heterogeneity in results. Variations in data quality, measurement techniques, and the timing of assessments could also influence the predictive accuracy of the models. Lastly, the use of diverse machine learning algorithms, with varying degrees of calibration and validation, might further explain the inconsistency in model performance across studies.

The predictive performance of the Support Vector Machine (SVM) was relatively low, indicating that it may not be the best choice for PSD prediction in the current dataset and model setup. However, SVM could demonstrate different performance in other studies or datasets, and future research should explore its applicability in various conditions. Similarly, the predictive performance of the biomarker group was relatively low, suggesting limited predictive ability in this study. Nevertheless, certain inflammatory and metabolism-related biomarkers showed predictive potential, and further research should validate their application value in PSD prediction. The risk of bias in several included studies is unclear, which may weaken the strength of the evidence.

## Conclusion

In conclusion, this meta-analysis highlights the promise of machine learning models, particularly neural networks, in predicting PSD, while also emphasizing the importance of functional, physical, and cognitive assessments as key data sources. The role of biomarkers, such as inflammatory and metabolic factors, also warrants further exploration as potential predictive tools. Moving forward, more robust and diverse studies are needed to refine machine learning models, identify novel biomarkers, and enhance the predictive accuracy and generalizability of PSD prediction tools in clinical settings. This is the first time that a meta-analysis has found varying results for PSD prediction depending on the models and data used, as previous studies have not conducted similar research.

## Data Availability

The original contributions presented in the study are included in the article/**Supplementary Material**. Further inquiries can be directed to the corresponding authors.

## Supplementary material

Supplementary Table 1 Details of the search strategy used for PubMed, Embase, Cochrane Library, and Web of Science. This table provides the list of keywords, MeSH terms, and Boolean operators applied for retrieving studies on PSD prediction models.

Supplementary Table 2 Summary of the included studies. This table includes study characteristics such as design, data sources, prediction models, and performance metrics (e.g., AUC, sensitivity, specificity).

Supplementary Table 3 Risk of bias assessment results for each included study based on the PROBAST tool. The table highlights individual ratings across four domains: participants, predictors, outcomes, and statistical analysis.
