# Supplementary material for: Clinical prediction models for post-stroke depression: a systematic review and meta-analysis
Source: Front Psychiatry. 2025 Dec 17;16:1629023. doi: 10.3389/fpsyt.2025.1629023 (PMC12753921; doi:10.3389/fpsyt.2025.1629023)
Supplement: Supplementary Table 1 — Details of the search strategy used for PubMed, Embase, Cochrane Library, and Web of Science. This table provides the list of keywords, MeSH terms, and Boolean operators applied for retrieving studies on PSD prediction models. [file Table1.docx]

## Table 1 Search strategy on Pubmed

| #1 | (Stroke[MeSH Terms] OR Cerebrovascular Accident[Title/Abstract] OR Ischemic Stroke[Title/Abstract] OR Hemorrhagic Stroke[Title/Abstract]) AND (Post-Stroke Depression[Title/Abstract] OR PSD[Title/Abstract] OR Stroke Depression[Title/Abstract] OR Depressive Disorder after Stroke[Title/Abstract]) |
| --- | --- |
| #2 | (Machine Learning[Title/Abstract] OR Artificial Intelligence[Title/Abstract] OR Deep Learning[Title/Abstract] OR Neural Networks[Title/Abstract] OR Supervised Learning[Title/Abstract] OR Unsupervised Learning[Title/Abstract] OR Random Forest[Title/Abstract] OR Support Vector Machine[Title/Abstract] OR Gradient Boosting[Title/Abstract] OR Decision Trees[Title/Abstract] OR Reinforcement Learning[Title/Abstract]) |
| #3 | (Prediction Model[Title/Abstract] OR Clinical Prediction Model[Title/Abstract] OR Risk Model[Title/Abstract] OR Prognostic Model[Title/Abstract] OR Predictive Models[Title/Abstract] OR Risk Assessment[Title/Abstract] OR Statistical Models[Title/Abstract] OR Prognosis[Title/Abstract] OR Model Development[Title/Abstract] OR Model Validation[Title/Abstract]) |
| #4 | #2 OR #3 |
| #5 | #1 AND #4 |
