# Supplementary material for: Clinical prediction models for post-stroke depression: a systematic review and meta-analysis
Source: Front Psychiatry. 2025 Dec 17;16:1629023. doi: 10.3389/fpsyt.2025.1629023 (PMC12753921; doi:10.3389/fpsyt.2025.1629023)
Supplement: Supplementary Table 2 — Summary of the included studies. This table includes study characteristics such as design, data sources, prediction models, and performance metrics (e.g., AUC, sensitivity, specificity). [file Table2.docx]

Table S2: The detail of included studies

| Author/Years  /Country | Number of patients | Predictive model | Model group | Data source | Outcome | Validation | Study Type |
| --- | --- | --- | --- | --- | --- | --- | --- |
| Fast L  2023  US | 309 | SVM-lin | SVM | Clinical variables, serological markers, MRI | Studies Depression Scale (CES-D) | Nested cross-validation | Retrospective  Cohort study |
|  |  | SVM-rbf | SVM | Clinical variables, serological markers, MRI |  |  |  |
|  |  | Gradient Boosting | Decision Tree | Clinical variables, serological markers, MRI |  |  |  |
| Gong J  2023  China | 2085 | Extreme gradient boosting | Decision Tree | Liver function test | Hamilton Depression Scale-17 | NA | Retrospective study |
|  | 2085 | Random forest | Decision Tree |  |  |  |  |
|  | 2085 | CatBoost | Decision Tree |  |  |  |  |
|  | 2085 | Gradient boosting decision tree | SVM |  |  |  |  |
|  | 2085 | SVM | Logistic Regression |  |  |  |  |
|  | 2085 | Logistic regression | Decision Tree |  |  |  |  |
| Hama S  2020  Japan | 274 | LLGMN, Log-Linearized Gaussian Mixture Network | Neural Network | Functional, physical, and cognitive tests | HADS Hospital Anxiety and Depression Scale | Cross-validation | Cross-sectional study |
|  |  | Stepwise multiple linear regression | Logistic Regression |  |  |  |  |
|  |  | Logistic regression | Logistic Regression |  |  |  |  |
|  |  | PLS regression | Logistic Regression |  |  |  |  |
| Ryu YH  2022  Korea | 75 | SVM | SVM | Functional, physical, and cognitive tests | Hamilton Rating Scale for Depression | Cross-validation. | Retrospective cohort study |
|  |  | KNN | K-nearest neighbor |  |  |  |  |
|  |  | RF | Decision Tree |  |  |  |  |
| Song SI  2022  Korea | 385 | XGBoost | Decision Tree | Functional, physical, and cognitive tests | Beck Depression Inventory | Cross-validation | Retrospective cohort study |
|  | 385 | CatBoost | Decision Tree |  |  |  |  |
|  |  | Light GBM | Decision Tree |  |  |  |  |
|  |  | Logistic regression | Logistic Regression |  |  |  |  |
| Zhang X  2023  US | 81 | Random forest | Decision Tree | SDHD | Hamilton Depression Rating Scale | Cross-validation | Retrospective cohort study |
|  |  | Random forest | Decision Tree | FERMT3 |  |  |  |
| Qiu X  2021  China | 503 | Artificial Neural Network | Neural Network | sleeping time CHD physical exercise BI score N dimension subjective support | Hamilton Depression Scale | Cross-validation | Prospective multicenter cohort study |
|  |  | Decision tree | Decision Tree |  |  |  |  |
| Chen YM  2023  China | 15366 | XGBoost | Decision Tree | Sociological data and Clinical data | Montgomery and Asberg Depression Rating Scale (MADRS) and  Hospital Anxiety and Depression Scale (HADS) | Cross-validation | Retrospective cohort study |
| de Man-van Ginkel JM  2013  Netherlands | 410 | Logistic Regression Model | Logistic Regression | Sociological data and Clinical data | Diagnostic and Statistical Manual of Mental Disorders | Cross-validation | Prospective multicenter cohort study |
| Cheng LS  2018  China | 259 | logistic regression analysis | Logistic Regression | C-reactive protein | Hamilton depression rating scale | NA | Prospective cohort study |
|  |  | logistic regression analysis | Logistic Regression | Sociological data and Clinical data |  |  |  |
|  |  | logistic regression analysis | Logistic Regression | FBG fasting blood glucose |  |  |  |
|  |  | logistic regression analysis | Logistic Regression | Homocystine |  |  |  |
|  |  | logistic regression analysis | Logistic Regression | C-reactive protein and Homocystine combined |  |  |  |
| Qiu HC  2018  China | 698 | binary logistic regression analysis | Logistic Regression | Insulin Resistance | Beck Depression Inventory Fast Screen | NA | Prospective cohort study |
|  |  | binary logistic regression analysis | Logistic Regression | Sociological data and Clinical data |  |  |  |
|  |  | binary logistic regression analysis | Logistic Regression | Insulin Resistance combined clinical data |  |  |  |
| Tu WJ  2018  China | 1205 | binary logistic regression analysis | Logistic Regression | Irisin | Hamilton Depression Rating Scale | NA | Prospective cohort study |
|  |  | binary logistic regression analysis | Logistic Regression | Sociological data and Clinical data |  |  |  |
|  |  | binary logistic regression analysis | Logistic Regression | Irisin and clinical data Combined |  |  |  |
| Luo S  2022  China | 202 | logistic regression analysis | Logistic Regression | Sociological data and Clinical data | Hamilton depression rating scale | NA | Prospective cohort study |
| Piechota M  2023  Poland | 96 | Logistic Regression | Logistic Regression | Gene Expression | Patient Health Questionnaire-9 | NA | Prospective cohort study |
| Luo S  2023  China | 206 | nomogram model | Logistic Regression | DNA methylation combined Clinical data | Hamilton depression rating scale | NA | Prospective cohort study |
| Li Z  2024  China | 374 | multivariate logistic regression model | Logistic Regression | Sociological data and Clinical data | Hamilton Depression Rating Scale (HAMD) | NA | Retrospective Cohort Study |
