# Supplementary material for: Clinical prediction models for post-stroke depression: a systematic review and meta-analysis
Source: Front Psychiatry. 2025 Dec 17;16:1629023. doi: 10.3389/fpsyt.2025.1629023 (PMC12753921; doi:10.3389/fpsyt.2025.1629023)
Supplement: Supplementary Table 3 — Risk of bias assessment results for each included study based on the PROBAST tool. The table highlights individual ratings across four domains: participants, predictors, outcomes, and statistical analysis. [file Table3.docx]

## PROBAST+AI MODEL EVALUATION

|  | Fast L  2023 | Gong J  2023 | Hama S  2020 | Ocei CW  2023 | Ryu YH  2022 | Song SI  2022 | Zhang X  2023 | Qiu X  2021 | Chen YM  2023 | *de Man*  *2013* | *Yue Y*  *2016* | *Cheng LS*  *2018* | *Qiu HC*  *2018* | *Tu WJ*  *2018* | *Liegey JS*  *2021* | Li G  2021 | Ladwig S  2022 | Luo S  2023 | Piechota M  2023 | *Luo S*  *2023* | Li Z  2024 |
| --- | --- | --- | --- | --- | --- | --- | --- | --- | --- | --- | --- | --- | --- | --- | --- | --- | --- | --- | --- | --- | --- |
| DOMAIN 1: Participants and data sources |  |  |  |  |  |  |  |  |  |  |  |  |  |  |  |  |  |  |  |  |  |
| A Risk of bias |  |  |  |  |  |  |  |  |  |  |  |  |  |  |  |  |  |  |  |  |  |
| 1.1 Were appropriate data sources used | Y | Y | Y | Y | Y | Y | Y | Y | Y | Y | Y | Y | Y | Y | Y | Y | Y | Y | Y | Y | Y |
| 1.2 Was an appropriate study design used? | Y | Y | Y | Y | Y | Y | Y | Y | Y | Y | Y | Y | Y | Y | Y | Y | Y | Y | Y | Y | Y |
| 1.3 Did the in- and exclusions of study participants result in a representative dataset? | Y | Y | Y | unclear | Y | Y | Y | Y | Y | Y | Y | Y | Y | Y | unclear | unclear | unclear | Y | Y | Y | Y |
| Risk of bias introduced by the selection of participants and data sources | low | low | low | Low | low | low | low | Unclear | low | low | Low | low | low | low | low | Y | unclear | Y | unclear | Y | Y |
| B. Applicability |  |  |  |  |  |  |  |  |  |  |  |  |  |  |  |  |  |  |  |  |  |
| Concern that the (data of the) included participants do not match the review question or the assessor’s intended use. | low | low | low | Unclear | low | low | low | low | low | low | Low | low | low | low | low | unclear | unclear | low | low | low | low |
| DOMAIN 2: Predictors |  |  |  |  |  |  |  |  |  |  |  |  |  |  |  |  |  |  |  |  |  |
| A. Risk of bias |  |  |  |  |  |  |  |  |  |  |  |  |  |  |  |  |  |  |  |  |  |
| 2.1 Were predictors defined and assessed in a similar way for all participants? | Y | Y | Y | Y | Y | Y | Y | PY | Y | Y | Y | Y | PY | Y | Y | Y | PY | Y | Y | Y | Y |
| 2.2 Was any pre-processing of predictors similar for all participants? | PY | Y | PY | Y | Y | Y | PY | PY | Y | Y | Y | Y | Y | Y | PY | Y | Y | Y | PY | Y | Y |
| 2.3 Were predictor assessments made without knowledge of outcome data? | Y | Y | Y | Y | Y | Y | Y | Y | Y | Y | Y | Y | Y | Y | Y | Y | Y | Y | Y | Y | Y |
| 2.4 Were the predictors included in the model available at the time the model was  intended to be used? | Y | Y | Y | Y | Y | Y | Y | PY | Y | Y | Y | NI | NI | NI | NI | NI | NI | NI | NI | NI | NI |
| Risk of bias introduced by the predictors or their assessment | low | low | low | Unclear | low | low | low | unclear | low | low | Low | unclear | low | unclear | unclear | low | unclear | unclear | low | low | low |
| B. Applicability |  |  |  |  |  |  |  |  |  |  |  |  |  |  |  |  |  |  |  |  |  |
| Concern that the definition, pre-processing, assessment, or timing of assessment of the  predictors in the model do not match the review question or the assessor’s intended  use | low | low | low | unclear | low | low | low | low | low | low | Low | low | low | low | low | low | low | low | low | low | low |
| DOMAIN 3: Outcome | low | low | low | Low | low | low | low | unclear | low | low | Low | low | low | low | low | low | unclear | unclear | unclear | unclear | low |
| A. Risk of bias |  |  |  |  |  |  |  |  |  |  |  |  |  |  |  |  |  |  |  |  |  |
| 3.1 Were outcomes defined and assessed appropriately? | Y | Y | Y | Y | Y | Y | Y | Y | Y | Y | Y | Y | Y | Y | Y | Y | Y | Y | Y | Y | Y |
| 3.2 Were outcomes defined and assessed in a similar way for all participants? | Y | Y | Y | Y | Y | Y | Y | Y | Y | Y | Y | Y | Y | Y | Y | Y | Y | PY | Y | PY | Y |
| 3.3 Were outcome assessments made without use or knowledge of predictor data? | Y | Y | Y | Y | Y | Y | Y | Y | Y | Y | Y | Y | Y | Y | Y | Y | Y | Y | Y | Y | Y |
| 3.4 Was the time interval between predictor assessment and outcome assessment  appropriate? | Y | Y | Y | Y | Y | Y | Y | Y | Y | Y | Y | Y | Y | Y | Y | Y | Y | Y | Y | Y | Y |
| Risk of bias introduced by the outcome or its determination | low | low | low | Unclear | low | low | low | low | low | low | unclear | low | low | low | low | low | low | low | low | low | low |
| B. Applicability | low | low | low | Unclear | low | low | low | low | low | low | Low | low | low | low | low | low | low | low | low | low | low |
| Concern that the outcome, its definition, assessment, or timing of assessment do not  match the review question or the assessor’s intended use | low | low | low | low | low | low | low | low | low | low | Low | low | low | low | unclear | unclear | unclear | low | low | low | low |
| DOMAIN 4: Analysis | low | low | low | Unclear | low | low | low | low | low | low | Low | low | low | low | Unclear | unclear | unclear | low | low | low | low |
| 4.1 Was model evaluation based on only apparent performance avoided? | Y | NI | Y | Y | Y | Y | Y | Y | Y | Y | Y | Y | Y | Y | Y | Y | Y | Y | Y | Y | Y |
| 4.2 Was there evidence that the sample size was reasonable? | PY | Y | PY | Y | Y | Y | Y | Y | Y | Y | PY | Y | Y | Y | Y | Y | PY | PY | PY | Y | Y |
| 4.3 Were participants with missing or censored data handled appropriately in  the analysis? | Y | Y | Y | Y | Y | Y | Y | Y | Y | Y | Y | Y | Y | Y | Y | Y | Y | Y | Y | Y | Y |
| 4.4 If methods to address class imbalance were used, was the evaluation done in  a dataset without imbalance correction? | Y | Y | Y | Y | NA | NA | Y | Y | Y | Y | Y | Y | Y | Y | Y | Y | Y | Y | Y | Y | Y |
| 4.5 If data splitting was done to create training and test datasets, was there  evidence that data leakage was avoided?* | Y | Y | Y | Y | Y | Y | Y | Y | Y | NA | NA | NA | NA | NA | NA | NA | NA | NA | NA | NA | NA |
| 4.6 If resampling methods were used to evaluate model performance, were all  model development steps replicated in the resampling process?* | NA | NA | NA | NA | NA | NA | NA | NA | NA | NA | NA | NA | NA | NA | NA | NA | NA | NA | NA | NA | NA |
| 4.7 Was the predictive performance of the model evaluated appropriately, e.g.,  calibration, discrimination, and net benefit? | Y | Y | Y | PY | PY | Y | Y | Y | PY | NA | NA | NA | NA | NA | NA | NA | NA | NA | NA | NA | NA |
| Risk of bias introduced by the analysis |  |  |  |  |  |  |  |  |  |  |  |  |  |  |  |  |  |  |  |  |  |
| RISK OF BIAS: | Low |  |  |  |  |  |  |  |  |  |  |  |  |  |  |  |  |  |  |  |  |
|  |  |  |  |  |  |  |  |  |  |  |  |  |  |  |  |  |  |  |  |  |  |
| OVERALL JUDGEMENT |  |  |  |  |  |  |  |  |  |  |  |  |  |  |  |  |  |  |  |  |  |
| RISK OF BIAS: | low | low | low | Unclear | low | low | low | unclear | low | low | unclear | low | low | low | Unclear | Unclear | Unclear | low | low | low | low |
| APPLICABILITY CONCERN: | low | low | low | Unclear | low | low | low | unclear | low | low | Low | low | low | low | low | unclear | low | low | low | low | low |
